# Amantadine Can Treat Blepharospasm in Levodopa Peak‐Dose Dyskinesia

**DOI:** 10.1002/mdc3.70220

**Published:** 2025-07-16

**Authors:** Francisco de Assis Aquino Gondim, Rodrigo Fagundes da Rosa, Francisco Duque de Paiva Giudice Junior, João Pedro de Oliveira Gouveia Marcotti, Ítalo Ramon de Araújo

**Affiliations:** ^1^ Neurology Division, Department of Internal Medicine Universidade Federal do Ceará Fortaleza State of Ceará Brazil; ^2^ Faculty of Medicine Universidade Federal do Ceará Fortaleza State of Ceará Brazil; ^3^ Neurology Division Hospital Universitário Walter Cantídio, Universidade Federal do Ceará Fortaleza State of Ceará Brazil

**Keywords:** amantadine, blepharospasm, peak dyskinesia, levodopa, case report

Most patients with Parkinson's disease (PD) develop motor fluctuations after few years of levodopa treatment.^1^ Levodopa peak‐dose dyskinesia is most frequently associated with chorea and limb dystonia.^2^ Blepharospasm has been rarely reported in the levodopa peak.^2^, ^3^ We describe a unique case of a PD patient with severe blepharospasm responsive to amantadine treatment.

On 3/2024, a 67‐year‐old man with a 19‐year PD history was transferred to our clinic. PD had started in 2005 and treated with multiple medications (Supplementary Material Table S1). After 10 years of PD treatment, he developed blepharospasm, treated with botulinum toxin and drug adjustments. During the Covid pandemic, he stopped taking several medications since in Brazil only levodopa can be purchased without a medical prescription. Over the last 2 years, approximately 1 h after taking one levodopa/benserazide (200/50) in the morning, he developed peak‐dose dyskinesia and severe blepharospasm (Video, Figure 1A). Blepharospasm was so debilitating that he stopped taking levodopa in the afternoon and nighttime, preferring to deal with rigidity, gait impairment and rest tremor (Video, Part I–II). He had no history of early dementia or autonomic dysfunction. His neurological exam (Video 1), revealed classic PD features: preserved cognitive status (no history of delirium or hallucinations), decreased blinking, facial hypomimia, bilateral palmomental and Myerson signs, preserved vertical eye movements, normal deep tendon reflexes, rest tremor, cogwheel rigidity and bradykinesia in the off‐period. We prescribed amantadine 100 mg TID and ordered Brain MRI to rule out Parkinson‐Plus disorders (more commonly linked to blepharospasm). Two months later, he reported major improvement of blepharospasm with amantadine without side effects (Video, Part III and Fig. 1B). He still exhibited important levodopa‐induced peak dyskinesia but with practically no blepharospasm (Fig. 1). Brain MRI did not disclose signs of Progressive Supranuclear Palsy (PSP) or Multiple System Atrophy (MSA). A levodopa challenge test (Fig. 1C) was conducted to understand the kinetics of the blepharospasm and dyskinesias. During his last appointment, blepharospasm worsened again after he run out of prescription briefly and subsequently improved after resuming amantadine.

**Figure 1:**
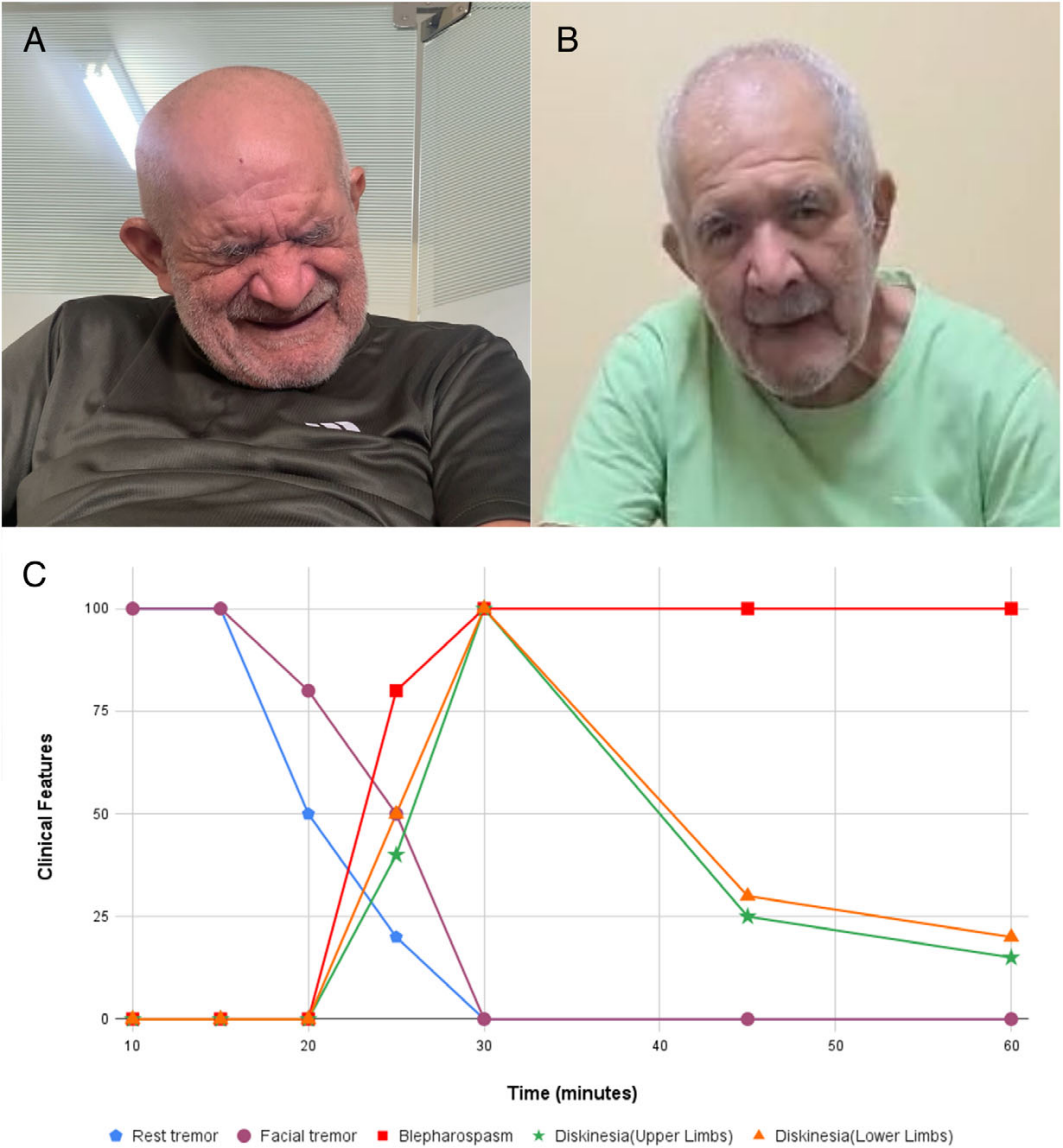
Effect of amantadine on levodopa‐induced peak blepharospasm. (A) Patient with peak blepharospasm 30 minutes after taking levodopa. (B) Patient shows a reduction in peak dose blepharospasm after taking levodopa in combination with amantadine. (C) Graph describing the course of dyskinesias, blepharospasm and parkinsonian features during the levodopa challenge test. After being deprived of amantadine for 7 days and not taking levodopa for 18 hours, we gave him a levodopa/benserazide (200/50) tablet (Fig. 1C, Video 1). Limb dyskinesias slowly started 25 minutes later in the legs and spread to the arms at 30 minutes. Approximately 2 minutes later, blepharospasm started and gradually increased to a maximum at 45 minutes. Generalized dyskinesias completely subsided at 45 minutes and severe blepharospasm persisted for the next hour.

**VIDEO 1:** The video shows a 67‐year‐old man with Parkinson's disease who has severe blepharospasm with peak‐dose levodopa dyskinesia (part II) and significant reduction in blepharospasm after adjunctive amantadine therapy (part III).

Peak‐dose dyskinesia occurs during the plateau of levodopa plasma levels and mainly consists of chorea, limb and/or trunk dystonia.^2^, ^3^ Amantadine is commonly employed to treat motor fluctuations and dyskinesias in PD.^1^ Amantadine can also be useful to treat unusual motor features in parkinson‐plus syndromes, eg, alien limb phenomenon.^4^

Eye‐related involuntary movements may be present in levodopa peak‐dose dyskinesia and are usually associated with movements in other parts of the body.^3^ Blepharospasm can be classified as a form of focal dystonia.^5^ It is more common in PSP (20–70%), although it can also occur in MSA and PD.^5^ Patients with blepharospasm may also have combined apraxia of eyelid opening.^5^ Blepharospasm in levodopa‐peak dyskinesia is a rare phenomenon in PD.^2^, ^3^, ^5^ Fan et al 2022 reported a PD patient treated with levodopa and pramipexole that developed blepharospasm in the peak‐dose period, that was attenuated by amantadine at the dose of 100 mg BID.^3^ Our patient differs from the description of Fan et al^3^: 1. He stopped taking PM levodopa to decrease blepharospasm (without additional medications). Second, blepharospasm was debilitating and present after a long PD history (dysregulation of dopamine receptors). Third, to our knowledge, this pattern of long‐lasting severe blepharospasm starting with the peak‐dose dyskinesias and persisting for at least 1 hour (leading to major impairment) has never been documented. Thus, our case is the first report to document a dramatic response of severe (debilitating) blepharospasm (present together with levodopa‐peak dyskinesia) to amantadine in a patient solely treated with levodopa.

## Author Roles

(1) Research Project: A. Conception, B. Organization, C. Execution. (2) Statistical Analysis: A. Design, B. Execution, C. Review and Critique. (3) Manuscript Preparation: A. Writing of the first draft, B. Review and Critique.

F.A.A.G: 1A, 1B,1C, 2A, 2B, 2C, 3A, 3B.

R.F.d.R: 1A, 1B,1C, 2A, 2B, 2C, 3A, 3B.

F.D.P.G.J: 1B,1C, 2B, 2C, 3A, 3B.

J.P.M: 1C, 2B, 2C.

I.R.d.A: 1C, 2B, 2C.

## Disclosures

**Ethical Compliance Statement:** Written informed consent for the publication of clinical details, images, and video was obtained from the patient. This retrospective case report is exempt from IRB approval per institutional policy from the Universidade Federal do Ceará. We confirm that we have read the Journal's position on issues involved in ethical publication and affirm that this work is consistent with those guidelines.

**Funding Sources and Conflicts of Interest:** The authors declare no conflicts of interest related to the research of this manuscript. No specific funding was received for this work.

**Financial Disclosures for the Previous 12 Months:** The authors declare that there are no additional disclosures to report.

## Supporting information


**Table S1.** List of medications used by the patient from the diagnosis of Parkinson's Disease to date.

## Data Availability

The data that support the findings of this study are available from the corresponding author upon reasonable request.
