# Supplementary material for: Amantadine Can Treat Blepharospasm in Levodopa Peak‐Dose Dyskinesia
Source: Mov Disord Clin Pract. 2025 Jul 16;12(12):2363–5. doi: 10.1002/mdc3.70220 (PMC12999024; doi:10.1002/mdc3.70220)
Supplement: Supplementary file 2 — Table S1. List of medications used by the patient from the diagnosis of Parkinson's Disease to date. [file MDC3-12-2363-s001.docx]

Supplementary material

**Table 1.** List of medications used by the patient from the diagnosis of Parkinson´s Disease to date*.

| **Year** | **Main Medications** | **Relevant Notes** |
| --- | --- | --- |
| 2007- 2015 | Different doses of Levodopa/Benserazide [100/25 (Prolopa BD ®) and 200/50 (Prolopa ®)]+Biperiden 2 mg BID (Akineton ®) | Initial treatment |
| 2016 | Pramipexole initiated and titrated up to 1 mg TID |  |
| 2017 | Different doses of Prolopa ® 200/50 (up to 5x/day), Pramipexole 1 mg TID, Amantadine 100 mg TID, Levodopa/Benserazide Extended Release 100/25 (Prolopa HBS®) at night | Progressive Blepharospasm |
| 2015-2017 | Botulinum toxin was used chronically to treat blepharospasm | The patient reported slight improvement in his blepharospasm. |
| 2018 | Prolopa BD ® 100/25 (up to 1.5 tablets 5x/day) or Prolopa ® 200/50 (up to QID), Pramipexole 1 mg TID, Amantadine 100 mg TID, Entacapone 200 mg up to 5x/day | First time the possibility of PSP was considered |
| 2019 | Prolopa® 200/50 (up to 5x/day), Pramipexole 1 mg TID, Amantadine 100 mg TID, Entacapone 200 mg up to 5x/day | Patient lost follow-up (December 2019) |
| 2020- 2024 | Prolopa ® (Levodopa/Benserazide 200/50 maximum TID) | In Brazil, only levodopa+benserazide or levodopa+carbidopa are sold without a medical prescription |
| 2024- 2025 | Prolopa ® (Levodopa/Benserazide 200/50) TID, Amantadine 100 mg TID |  |

*Due to financial constraints and variable and uncertainly therapeutic adhesion its very likely that the patient did not follow exactly prescriptions from first two groups of neurologists
